# Reinstitution of Mechanical Ventilation within 14 Days as a Poor Predictor in Prolonged Mechanical Ventilation Patients following Successful Weaning

**DOI:** 10.1100/2012/957126

**Published:** 2012-07-31

**Authors:** Mei-Lien Tu, Ching-Wan Tseng, Yuh Chyn Tsai, Chin-Chou Wang, Chia-Cheng Tseng, Meng-Chih Lin, Wen-Feng Fang, Yung-Che Chen, Shih-Feng Liu

**Affiliations:** ^1^Division of Pulmonary & Critical Care Medicine, Department of Internal Medicine, Kaohsiung Chang Gung Memorial Hospital, Chang Gung University College of Medicine, Kaohsiung, Taiwan; ^2^Department of Respiratory Therapy, Kaohsiung Chang Gung Memorial Hospital, Chang Gung University College of Medicine, Kaohsiung, Taiwan

## Abstract

Although many parameters were investigated about weaning and mortality in critical patients in intensive units, no studies have yet investigated predictors in prolonged mechanical ventilation (PMV) patients following successful weaning. A cohort of 142 consecutive PMV patients with successful weaning in our respiratory care center was enrolled in this study. Successful weaning is defined as a patient having smooth respiration for more than 5 days after weaning. The results showed as follows: twenty-seven patients (19%) had the reinstitution within 14 days, and 115 patients (81%) had the reinstitution beyond 14 days. Renal disease RIFLE-LE was associated with the reinstitution within 14 days (*P* = 0.006). One year mortality rates showed significant difference between the two groups (85.2% in the reinstitution within 14 days group versus 53.1% in the reinstitution beyond 14 days; *P* < 0.001). Kaplan-Meier analysis showed that age ≥70 years (*P* = 0.04), ESRD (*P* = 0.02), and the reinstitution within 14 days (*P* < 0.001) were associated with one-year mortality. Cox proportional hazards regression model showed that only the reinstitution within 14 days was the independent predictor for mortality (*P* < 0.001). In conclusion, the reinstitution within 14 days was a poor predictor for PMV patients after successful weaning.

## 1. Introduction

In recent years, advanced intensive care has prolonged survival rates in patients with severe illness than the past. Patients with prolonged mechanical ventilation (PMV) generally suffer from a “chronic critical illness” characterized by a variety of significant, characteristic derangements of metabolism and neuroendocrine, neuropsychiatric, and immunologic functions [1], and these patients often become ventilator-dependent. Without facilities for postintensive care, these patients would require extended stays in intensive care units (ICUs). The cost of such patients on prolonged mechanical ventilation (PMV) could account for approximately 5–20% of the total budget and resources earmarked for intensive care by the Taiwan Central Government. Mechanical ventilation has been recognized as one of the major critical care treatment modalities that can be executed beyond the confines of the ICU, thus establishing a critical care continuum in step-down units, noninvasive respiratory care units, and long-term care hospitals [2–10]. To increase the availability of ICU beds for acute illness patients and to improve successful weaning rates for chronic respiratory failure patients, the National Health Insurance Bureau of Taiwan also developed the integrated delivery system (IDS) in 1990. Since then, most PMV patients with exceeding 21 days were sent to respiratory care center (RCC) for further management.

Although many attempts have been made to demonstrate the importance of the integrated step-down care system for patients on long-term mechanical ventilators [2–10], and to determine predictive weaning factors for PMV patients [11–13], the predictors and outcomes of PMV patients following successful weaning have not been widely studied. Furthermore, although many parameters related to weaning and mortality were investigated in critical patients in intensive unit [14–16], we do not know whether these parameters are universally validated as scientific evidence-based predictors for such PMV patients following successful weaning. This study aimed to investigate prognostic factors for one-year mortality in these patients and compare the clinical characteristics between the reinstitution of mechanical ventilation within 14 days and beyond 14 days.

## 2. Material and Methods

### 2.1. Design, Setting, and Samples

This study was approved by the Institutional Review Board (IRB) of our hospital.

A cohort of consecutive PMV patients with successful weaning during a period was enrolled for the investigation of prognostic factors for one-year mortality and the clinical characteristics between the reinstitution of mechanical ventilation within 14 days and beyond 14 days. The setting was from RCC in Kaohsiung Chang Gung Memorial Hospital, a 2300-bed facility serving as a primary care and tertiary referral center in Taiwan, between November 2004 and June 2005, consecutive 142 PMV patients who were weaned successfully from ventilators in the RCC were recruited into this study. PMV is defined as mechanical ventilation ≥21 d. Successful weaning is defined as a patient having spontaneous respiration for more than 5 days without requiring mechanical ventilator support. Persistent acute renal failure (loss) is defined as need for renal replacement therapy for more than 4 weeks, whereas ESRD is defined as need for dialysis for longer than 3 months by the RIFLE definition [17]. After enrollment, the mortalities of these patients were followed up from chart records and computer information. If patients lost followup from our hospital, the research assistant would communicate with the patients or family members to get the information about mortality by telephone interview. The prognostic factors for survival in these PMV patients with successful weaning were also analyzed. Variables included age, gender, acute physiology and chronic health evaluation II score (APACHE II score), patient source, consciousness status, the initial causes of respiratory failure, tracheostomy, length of stay in RCC, MV duration, rapid shallow breathing index (RSBI), blood chemistry, hemogram, and underlying diseases.

### 2.2. Statistical Analysis

All data were analyzed by SPSS 11.5, the Statistical Program for the Social Science of the University of Michigan, Microsoft Corporation, Redmond, USA. Continuous variables are presented as mean ± SD, and discrete variables are expressed as numbers per study population (%). Univariate analysis was performed using Student's* t*-test (for quantitative variables of normal distribution) or chi-square or Fisher's exact tests (for qualitative variables). Multivariate analysis was performed by binary logistic regression with forward conditional method. The Cox proportional hazards regression model was used in univariate and multivariate analyses to identify the most significant prognostic factors for survival and to calculate hazards ratios (HRs) of death and 95% confidence intervals for the ratio. Survival was estimated with Kaplan-Meier method using log-rank test. A *P* value <0.05 was considered statistically significant.

## 3. Results

**Figure 1:**
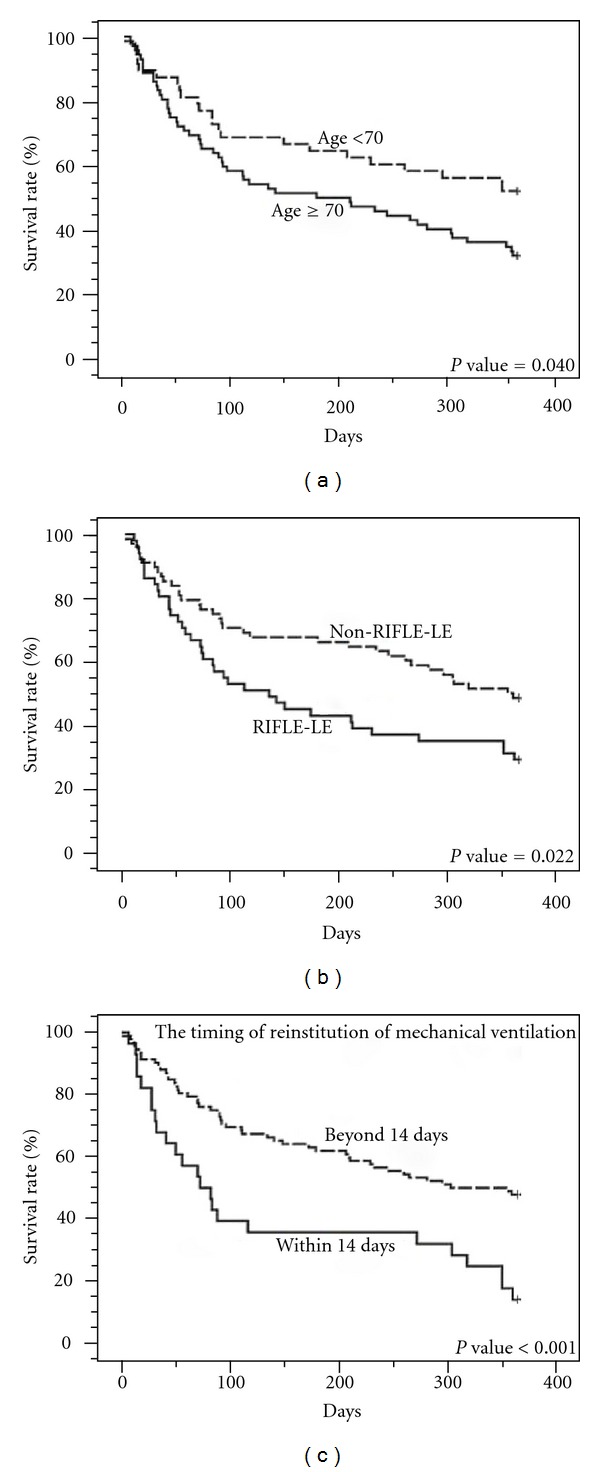
Kaplan-Meier analysis of prognostic factors for 1-year cumulative survival in 121 prolonged mechanical ventilation patients following successful weaning: age <70 versus ≥70 years (a), RIFLE-LE versus non-RIFLE-LE (b), and the reinstitution of mechanical ventilation within 14 days versus beyond (c).

A total of 142 PMV patients were enrolled in this study, and their characteristics were in Table 1. Twenty-seven patients (19%) had the reinstitution within 14 days, and 115 patients (81%) had the reinstitution beyond 14 days. Univariate analysis showed that mechanical ventilation duration and RIFLE-LE classification had significant differences between the two groups. However, multivariate analysis showed that RIFLE-LE classification was associated with the reinstitution within 14 days (*P* = 0.006). Kaplan-Meier analysis showed age ≥70 years (*P* = 0.04), RIFLE-LE classification (*P* = 0.02), and the reinstitution within 14 day (*P* < 0.001) were associated with one-year survival (Figure 1). Gender, patient transfer origin, consciousness status, tracheostomy, RCC stay duration, MV duration, RSBI, APACHE II score, blood chemistry, hemogram, and most underlying diseases were not associated with 1-year mortality. Cox proportional hazards regression model showed that reinstitution within 14 days was the independent prognostic factor for mortality (*P* < 0.001) (Table 2). Seventy-four patients (61%) died in one year in this study. Of the 27 patients with the reinstitution within 14 days, 2 patients lost followup; 23 patients (85.2%) died within 1 year. In contrast, of the 115 patients with reinstitution beyond 14 days, 19 patients lost followup; 51 patients (53.1%) died within 1 year (*P* < 0.001). Of 21 lost followup patients, 19 patients could not be contacted by telephone, and 2 patients' family refused cooperation.

## 4. Discussion

The main finding of this study demonstrated that about 60% of all PMV patients after successful weaning died in one-year and these patients with the reinstitution within 14 days had higher one year mortality rate up to 85.2%. Although more than 60% of PMV patients were successfully weaned from our RCC, these PMV patients after successful weaning with the reinstitution within 14 days implied an ominous sign for prognosis. Clinical physician should explain the unfavorable outcomes to family and attempt to reduce the intensity of care.

Renal failure requiring hemodialysis combined with ventilatory support in the post-ICU setting was a predictor of poor outcome [12, 18, 19]. In addition, a recent cohort study showed that with the use of biocompatible hemodialysis membranes and more frequent dialysis, 29% of 80 patients with severe renal dysfunction were liberated from PMV [12]. Similarly, our study showed that RIFLE-LE classification is also associated with the reinstitution within 14 days. The reasons may be multiple. RIFLE-LE classification is frequently associated with underlying diabetic mellitus in Taiwan and subsequent cardiovascular complications, frequent fluid and electrolyte imbalance, as well as some toxic metabolic byproducts which may need more frequent hemodialysis and better hemodialysis membranes to remove. APACHE scores [14] and RBSI [15, 16] are considered to be very significant predictors of successful ICU extubation in the past decade, but they were not accepted as scientific evidence-based predictors of PMV [20, 21]. Serum albumin level was reported to be a good predictor for post-ICU weaning [13]. However, our study showed that APACHE scores, RBSI, or serum albumin were not the prognostic factors for PMV patients after successful weaning.

We estimated an about 40% 1-year survival rate in all PMV patients included in this study. However, 14.8% 1-year survival in the reinstitution within 14 days patients, 31% 1-year survival in age ≥70 years patients, and 29% 1-year survival in RIFLE-LE classification patients. Scheinhorn et al. reviewed previous data and found survival rates for 1 or more years after discharge following PMV to be in the 50% range at best [8]. Carson and colleagues reported a 23% 1-year survival in 133 PMV patients [22]. 14.8% 1-year survival in the reinstitution within 14 days is less than those of related reports. Obviously, the reinstitution within 14 days in PMV patients after weaning is very ominous sign.

 Old age is also a very important predictor for PMV patients [8, 23–26]. Although the old age is not a significant predictor in PMV patients after successful weaning in our study, 31% 1-year survival in age ≥70 years patients is still less than the average survival rate 39%. We reviewed the related papers. The premorbid functional status and age analysis for PMV patients showed that younger and more independent patients had a better mortality (56%), and older and more dependent patients had 95% mortality at 1 year [8]. The incremental costs per quality-adjusted life-year increased by prolonged mechanical ventilation provision at age ≥68 years, and the predicted 1-year mortality was >50% [25]. The severity of illness at the time of admission to the ICU and the prehospitalization functional status had a significant association with the short-term mortality rate, whereas age and comorbidities were related to long-term mortality [26]. Epstein et al. also reported that age ≥65 years was the independent predictors of death after ICU discharge [21]. 

## 5. Limitation

This study has some limitations. After successful weaning from PMV, the final destinations of these PMV patients discharge were different. Different destinations would provide different quality of care giving, and the mortality may differ. Another, we had higher percentage of patients who lost follow-up and this might influence mortality stastics. However, one-year mortality rate had significant difference between the two groups (85.2% in the reinstitution within 14 days group versus 53.1% in the reinstitution beyond 14 days). We believe that the results should be no significant change even if the information of all cases is available.

## 6. Conclusion

The reinstitution within 14 days was a poor predictor for PMV patients after successful weaning. Physician should highlight such condition.

## Figures and Tables

**Table 1:** Clinical characteristics of 142 prolonged mechanical ventilation patients following weaning and comparison between the reinstitution within 14 days and beyond 14 days.

Variables	Total subjects *N* = 142	Reinstitution within 14 days *N* = 27	Reinstitution beyond 14 days *N* = 115	*P*
Age (year)	68.7 ± 15.1	71.0 ± 12.9	68.1 ± 15.6	0.38
Gender (M/F)	79/63	15/12	64/51	0.99
Transfer origin MICU/SICU*	113/29	19/8	93/22	0.39

Initial causes of ARF				
ARDS (%)	27 (19.0)	5 (18.5)	22 (19.1)	0.95
CHF (%)	35 (24.6)	5 (18.5)	30 (24)	0.65
COPD (%)	16 (11.3)	3 (11.1)	13 (11.3)	0.98
Neurologic disorder (%)	13 (9.2)	3 (11.1)	11 (8)	0.62
After operation (%)	29 (20.4)	5 (18.5)	24 (20.8)	0.89
Other (%)	22 (15.5)	4 (14.8)	18 (15.6)	0.89
Tracheostomy (%)	56 (39.4)	14(52)	43 (37)	0.21
Coma (%)	65 (45.8)	16 (61)	48 (42)	0.12
Length of stay in RCC (days)	17.0 ± 9.9	18.1 ± 10.7	16.7 ± 9.6	0.81
MV duration (days)	34.7 ± 15.6	40.2 ± 19.4	33.5 ± 14.3	0.04
RSBI	100.2 ± 46.3	109.6 ± 53.2	96.9 ± 43.7	0.14
APACHE II scores	16.3 ± 7.3	18.7 ± 7.4	15.7 ± 7.2	0.053

Underlying disease				
Diabetes mellitus (%)	56 (39.4)	11 (41)	45 (39)	0.84
Hypertension (%)	91 (64.1)	18 (67)	72 (63)	0.74
RIFLE-LE^*∧*^ (%)	60 (42.3)	18(67)	15 (37)	0.005
Stroke (%)	47 (33.1)	6 (22)	41 (36)	0.18
Liver cirrhosis (%)	15 (10.6)	4 (15)	11 (10)	0.19
COPD (%)	73 (51.4)	9 (33)	58 (50)	0.27

Blood cell count				
WBC (10^3^/cmm)	8722 ± 816	8.8 ± 3.1	8.6 ± 4.0	0.92
Hemoglobin (g/dL)	10.6 ± 1.0	10.5 ± 0.9	10.7 ± 1.1	0.58
Hematocrit (%)	32.8 ± 3.5	31.9 ± 2.3	33.6 ± 4.4	0.30
Platelet (10^4^/cmm)	19.0 ± 1.9	17.5 ± 5.6	20.5 ± 9.5	0.48

Blood chemistry				
Na (meq/L)	135 ± 2.3	136 ± 1.8	135 ± 2.6	0.34
K (meq/L)	4.1 ± 0.4	4.0 ± 0.6	4.2 ± 0.3	0.44
BUN (meq/L)	47.6 ± 11.0	53.06 ± 20.0	42.9 ± 12.3	0.66
CR (mg/L)	1.9 ± 0.5	2.2 ± 0.9	1.6 ± 0.6	0.53
Albumin (g/dL)	2.3 ± 0.6	2.4 ± 0.5	2.2 ± 0.7	0.32

^∧^Classification of renal diseases by the RIFLE criteria (acronym indicating risk of renal dysfunction, injury to the kidney, failure of kidney function, loss of kidney function, and end-stage kidney disease). MICU/SICU: medical intensive care unit/surgical intensive care unit.

**Table 2:** Prognostic factors of 1-year mortality in prolonged mechanical ventilation patients with successful weaning by univariate and multivariate analyses.

Variables		*P*
Age (year)	<70 versus ≥70	0.04
Gender (M/F)	Male versus female	0.66
Transfer origin	MICU versus SICU	0.16
Tracheostomy	Yes versus no	0.50
Coma	Yes versus no	0.34
Stay duration in RCC (D)	<21 versus ≥21	0.13
MV duration (D)	<50 versus ≥50	0.80
RSBI	<105 versus ≥105	0.49
APACHE II scores	<25 versus ≥25	0.55
the reinstitution date	Within 14 days versus beyond	<0.001*

Underlying disease		
Diabetes mellitus	Yes versus no	0.65
Hypertension	Yes versus no	0.81
RIFLE-LE^∧^	Yes versus no	0.02
Stroke	Yes versus no	0.28
Liver cirrhosis	Yes versus no	0.89
COPD (%)	Yes versus no	0.50

*Cox proportional hazards regression model, *P* < 0.001; 95% CI = 1.64–4.51; HR = 2.72.

^∧^Classification of renal diseases by the RIFLE criteria (acronym indicating risk of renal dysfunction, injury to the kidney, failure of kidney function, loss of kidney function, and end-stage kidney disease).

## List of Abbreviations

PMV: Prolonged mechanical ventilation
ESRD: End-stage renal disease
RCC: Respiratory care center
ICUs: Intensive care units
IDS: Integrated delivery system
RSBI: Rapid shallow breathing index,
APACHE II: Acute physiology and chronic health evaluation II
RIFLE: Risk of renal dysfunction, injury to the kidney, failure of kidney function, loss of kidney function, and end-stage kidney disease.
