# Erratum to “Reinstitution of Mechanical Ventilation within 14 Days as a Poor Predictor in Prolonged Mechanical Ventilation Patients following Successful Weaning”

**DOI:** 10.1155/2013/402140

**Published:** 2013-09-09

**Authors:** Mei-Lien Tu, Ching-Wan Tseng, Yuh Chyn Tsai, Chin-Chou Wang, Chia-Cheng Tseng, Meng-Chih Lin, Wen-Feng Fang, Yung-Che Chen, Shih-Feng Liu

**Affiliations:** ^1^Department of Respiratory Therapy, Kaohsiung Chang Gung Memorial Hospital, Chang Gung University College of Medicine, Kaohsiung, Taiwan; ^2^Chang Gung University of Science and Technology, Taiwan; ^3^Division of Pulmonary & Critical Care Medicine, Department of Internal Medicine, Kaohsiung Chang Gung Memorial Hospital, Chang Gung University College of Medicine, 123, Ta-Pei Road, Niao-Sung Hsiang, Kaohsiung Hsien, Taiwan

In the paper titled “Reinstitution of Mechanical Ventilation within 14 Days as a Poor Predictor in Prolonged Mechanical Ventilation Patients following Successful Weaning,” the affiliations of the authors should be corrected as shown above.

